# The Use of a Novel Heart Failure Agent in the Treatment of Pregnancy-Associated Cardiomyopathy

**DOI:** 10.1155/2017/9561405

**Published:** 2017-08-14

**Authors:** Vamsi C. Gaddipati, Aarti A. Patel, Adam J. Cohen

**Affiliations:** Department of Cardiovascular Sciences, Morsani College of Medicine, University of South Florida, Tampa, FL, USA

## Abstract

Peripartum cardiomyopathy is an uncommon, pregnancy-related form of dilated cardiomyopathy that is associated with development of new-onset left ventricular dysfunction. Its etiology is presently unknown, but current standard of care involves the use of typical drug therapy for the treatment of heart failure. Pregnancy-associated cardiomyopathy (PACM) is a similar condition that refers to patients who develop such symptoms prior to the last month of pregnancy. We report the case of a nulliparous Caucasian female who develops early, severe PACM during her first pregnancy with postpartum persistence of New York Heart Association class II-III symptoms despite medical therapy. The use of the novel heart failure agent, sacubitril/valsartan (Entresto), is initiated with near-complete resolution of her symptoms.

## 1. Introduction

Peripartum cardiomyopathy (PPCM) is an uncommon pathology for pregnant women that can produce severe left ventricular (LV) dysfunction. Its diagnosis may be obfuscated as many symptoms are identical to those of normal pregnancy; accordingly, practitioners must have a high index of suspicion given the potentially fatal prognosis. Current standard of care involves treatment with the typical medication regimen for other forms of heart failure (HF). Ahead, we report the case of a 24-year-old Caucasian female who develops severe biventricular dysfunction early in her first pregnancy. Although her HF persists during the postpartum period, we describe the usage of a novel heart failure agent, sacubitril/valsartan (Entresto), to near-complete resolution of her symptoms.

## 2. Case Presentation

A 24-year-old Caucasian female presented to the Emergency Department at 17 weeks and 3 days of gestation of her first pregnancy with a two-month history of worsening dyspnea and lower extremity swelling. She noted progressively worsening symptoms over the prior two months to the point where she was unable to lie flat. She had no past medical history, and initial vital signs were notable for HR of 129, BP 117/85, RR 22, and SpO2 of 100% on room air. Physical exam demonstrated bilateral lower extremity pitting edema to the ankles, S3 gallop, and elevated jugular venous pressure. Initial laboratory values were remarkable for a mild transaminitis (AST 113 IU/L, ALT 189 IU/L) and a B-natriuretic peptide (BNP) of 1191 pg/mL. Presenting electrocardiogram revealed sinus tachycardia with left axis deviation, and computed tomography pulmonary angiogram was negative for pulmonary embolism. Transthoracic echocardiogram was pertinent for LV ejection fraction (EF) of 15–20% with severe global hypokinesis, grade 3-4 diastolic dysfunction, severe right ventricular dilatation and systolic dysfunction, moderate mitral and tricuspid regurgitation, and elevated pulmonary artery systolic pressure of 70 mmHg. Accordingly, she was admitted to the Cardiac Intensive Care Unit for management of new-onset biventricular HF.

Concurrent with aggressive diuresis, continuation of pregnancy was discussed extensively with the patient in tandem with the Obstetrics service. After electing to proceed, she was initiated on furosemide, hydralazine, isosorbide dinitrate (ISDN), and carvedilol. Work-up of the cardiomyopathy, unfortunately, did not reveal a clear etiology as viral serologies were negative and cardiac magnetic resonance imaging (MRI) demonstrated no evidence of edema, inflammation, or other alternative explanation. Following this admission, the patient was discharged home in stable condition and seen in clinic weekly with multiple repeat echocardiograms not demonstrating any significant improvement. Attempts to uptitrate her medical therapy were limited by New York Heart Association (NYHA) class II-III HF and symptomatic hypotension.

She presented for elective Caesarian section at 30 weeks and 3 days of gestation, which was completed without perioperative complication. Her recovery, however, was extended due to postoperative respiratory failure and decompensation necessitating Swan-Ganz catheter placement and transient inotropic support with dobutamine in conjunction with nitroprusside infusion. Cautious weaning led to discharge on carvedilol and enalapril (and cessation of hydralazine and ISDN).

Afterwards, she unfortunately reported continued fatigue and lethargy. Although her HF clinically appeared compensated, repeat echocardiography performed approximately two months following delivery was again without improvement. The Seattle Heart Failure Model calculator estimated a mean survival of approximately 9 years, and her case was referred to Transplant Cardiology for further evaluation and early establishment. Genetic testing for sarcomere mutations was performed and again did not evince clear alternative etiology to the cardiomyopathy.

**Figure 1:**
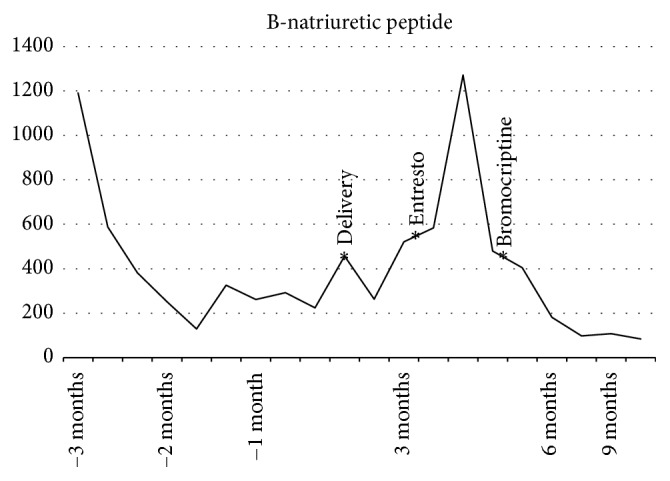
Progression of BNP levels during the peripartum course.

Given her refractory symptoms, the decision was made to attempt initiation of Entresto. Despite an initial HF exacerbation during the transition process that necessitated brief hospital admission for diuresis, the patient reported increased energy level, no further episodes of lightheadedness or dizziness, and minimal dyspnea within approximately one week. Though blood pressure in the clinic setting appeared similar to before, she reported improved ambulatory pressures with no longer needing to hold doses of her medications for hypotension. Titration of medications and BNP trend are reported in Table 1 and Figure 1.

She has done well thereafter and continues to be closely followed with cautious titration to optimal medical therapy. No further symptoms have been reported in 15 months of follow-up and LV EF has improved to 35–40% with resolution of right ventricular dysfunction.

## 3. Discussion

In the presentation described above, our patient would be categorized as pregnancy-associated cardiomyopathy (PACM) as she was diagnosed early in pregnancy. Though this disease process is not as well studied, it has been suggested to be phenotypically identical to PPCM and without clinical differences in presentation, management, or prognosis [1]. Accordingly, the two will herein be considered synonymous.

During pregnancy, hemodynamic and hormonal changes affect cardiac output, vascular relaxation, and thrombogenesis in addition to a variety of systemic metabolic parameters [2]. These changes may manifest into a variety of symptoms—fatigue, dyspnea, edema, chest pain, and so forth—that can be difficult to distinguish from the pathologic presentation of PPCM. Accordingly, this first challenge of diagnosis is paramount and requires a thorough history and physical examination in conjunction with high index of suspicion, biochemical laboratory markers, and imaging techniques. Criteria for diagnosis itself include the following: (1) development of HF in the last month of delivery or within 5 months of delivery; (2) the absence of a determinable etiology for HF; (3) absence of demonstrable heart disease before the last month of pregnancy; (4) LV systolic dysfunction demonstrated by LVEF < 45%, fractional shortening < 30%, or both [3].

Within the United States, the incidence of PPCM has been approximated at 0.3%, though with higher rates amongst the African American ethnicity [3]. In addition, it occurs with greater incidence in women with increased age, multiparity, and preeclampsia [4]. Familial clustering of PPCM has been noted, and ongoing studies suggest a potential genetic component to the development of this pathology [5, 6]. For this reason, there is a suggestion that the disorder is a subset of dilated cardiomyopathies that are evinced during the partum period. Potential pathogenetic basis has been theorized as abnormal autoimmune response, apoptosis, impaired cardiovascular microvasculature, systemic angiogenic imbalance, and oxidative stress [4, 6]. Given the discrepancy in manifestation and prognosis, however, the disease process is likely an interaction between multiple intrinsic mechanisms.

With known potentially fatal implications, outcome and prognosis remain an important focus of study. The Investigations of Pregnancy-Associated Cardiomyopathy study demonstrated that the majority of women (72%) recover EF, particularly those without significant remodeling (LV end-diastolic diameter < 6.0 cm) or severe LV dysfunction (LV EF ≥ 30%) [7]. Poorer outcomes have been associated with increased LV end-diastolic diameter, systolic blood pressure < 110 mmHg, and heart rates > 100 [8]. This latter subgroup was largely unable to tolerate goal dosages of beta blocker and angiotensin converting enzyme inhibitor (ACE-i) therapy. Cardiac MRI, as well, is a burgeoning field for the assessment of cardiac structure and function that has been suggested to display some prognostic implication in the setting of PPCM. The presence of late gadolinium enhancement may be associated with adverse cardiovascular outcomes and is congruent with higher relapse rates of heart failure in patients with persistent low EF [9]. Its usage during pregnancy, however, has been recommended by the American College of Radiology only if essential [3].

Current treatment is yet an evolving field, but standard of care is analogous to management of other types of HF (i.e., includes low-salt diet, fluid restriction, blood pressure control, loop diuretics, ACE-i or angiotensin receptor blocker therapy, beta blockade, nitrates, and hydralazine) [10]. Anticoagulation, defibrillators, and advanced therapies (mechanical assist devices or heart transplantation) are as well approached in a similar manner [11]. The efficacy of experimental therapies such as pentoxifylline and immunoglobulin is equivocal and remains under further investigation [3, 10, 11]. A promising current contemporary agent is bromocriptine, which prolongs prolactin inhibition and thereby reduces its antiangiogenic and proapoptotic effects [11, 12]. Early initiation in conjunction with typical medical therapy may improve prognosis in pilot studies and studies of HF recurrence in repeat pregnancies [13], but a greater level of evidence is still required prior to widespread adoption.

In our case above, we elected to proceed with initiation of a novel HF agent, Entresto, on the basis of results from the PARADIGM-HF trial [14]. While bromocriptine was as well provided, its introduction followed reported improvement in symptoms; furthermore, the dosage was not titrated to that used in the above investigations. It would appear that the driving factor for enhanced quality of life in this example was Entresto, suggesting a potential benefit in HF not limited to the archetypal presentation.

## 4. Conclusion

PPCM and PACM are potentially fatal conditions that require timely identification and treatment given their long-term sequelae and prognostic implications. While the usage of standard drug therapy for HF is the gold standard at present, novel agents may provide symptom relief in a subset of patients who are unable to tolerate the typical regimen. Further studies are needed to better ascertain optimal management of these high-risk patients.

## Figures and Tables

**Table 1:** Vital signs and medication regimen in the postpartum period.

Postpartum	SBP	DBP	HR	Medications
0 months	94	65	102	Carvedilol 6.25 mg BID, hydralazine 75-125-75 mg, isosorbide dinitrate 20 mg TID
1 month	95	65	94	Carvedilol 3.125 mg BID, enalapril 2.5 mg BID
2 months	100	70	109	Carvedilol 3.125 mg BID, enalapril 5 mg BID
3 months	96	72	103	Carvedilol 3.125 mg BID, enalapril 5 mg BID
4 months	92	65	90	Carvedilol 6.25 mg BID, Entresto 49-51 mg BID
5 months	90	60	77	Carvedilol 6.25 mg BID, Entresto 97-103 mg BID, bromocriptine 1.25 mg
6 months	94	61	77	Carvedilol 9.375 mg BID, Entresto 97-103 mg BID, bromocriptine 1.25 mg, eplerenone 25 mg
9 months	98	67	65	Carvedilol 9.375 mg BID, Entresto 97-103 mg BID, bromocriptine 1.25 mg, eplerenone 25 mg
12 months	112	77	81	Carvedilol 12.5 mg BID, Entresto 97-103 mg BID, bromocriptine 1.25 mg, eplerenone 25 mg
15 months	102	69	70	Carvedilol 12.5 mg BID, Entresto 97-103 mg BID, bromocriptine 1.25 mg, eplerenone 25 mg

## References

[1] Elkayam U., Akhter M. W., Singh H. (2005). Pregnancy-associated cardiomyopathy: clinical characteristics and a comparison between early and late presentation. *Circulation*.

[2] Hilfiker-Kleiner D., Haghikia A., Nonhoff J., Bauersachs J. (2015). Peripartum cardiomyopathy: Current management and future perspectives. *European Heart Journal*.

[3] Elkayam U. (2011). Clinical characteristics of peripartum cardiomyopathy in the United States: diagnosis, prognosis, and management. *Journal of the American College of Cardiology*.

[4] Patten I. S., Rana S., Shahul S. (2012). Cardiac angiogenic imbalance leads to peripartum cardiomyopathy. *Nature*.

[5] Morales A., Painter T., Li R. (2010). Rare variant mutations in pregnancy-associated or peripartum cardiomyopathy. *Circulation*.

[6] van Spaendonck-Zwarts K. Y., Posafalvi A., van den Berg M. P. (2014). Titin gene mutations are common in families with both peripartum cardiomyopathy and dilated cardiomyopathy. *European Heart Journal*.

[7] McNamara D. M., Elkayam U., Alharethi R. (2015). Clinical outcomes for peripartum cardiomyopathy in North America: results of the IPAC study (Investigations of Pregnancy-Associated Cardiomyopathy). *Journal of the American College of Cardiology*.

[8] Libhaber E., Sliwa K., Bachelier K., Lamont K., Böhm M. (2015). Low systolic blood pressure and high resting heart rate as predictors of outcome in patients with peripartum cardiomyopathy. *International Journal of Cardiology*.

[9] Arora N. P., Mohamad T., Mahajan N. (2014). Cardiac magnetic resonance imaging in peripartum cardiomyopathy. *American Journal of the Medical Sciences*.

[10] Garg J., Palaniswamy C., Lanier G. M. (2015). Peripartum cardiomyopathy: Definition, incidence, etiopathogenesis, diagnosis, and management. *Cardiology in Review*.

[11] Bouabdallaoui N., Mouquet F., Lebreton G., Demondion P., Le Jemtel T. H., Ennezat P. V. (2016). Current knowledge and recent development on management of peripartum cardiomyopathy. *European Heart Journal: Acute Cardiovascular Care*.

[12] Haghikia A., Podewski E., Libhaber E. (2013). Phenotyping and outcome on contemporary management in a German cohort of patients with peripartum cardiomyopathy. *Basic Research in Cardiology*.

[13] Hilfiker-Kleiner D., Haghikia A., Masuko D. (2017). Outcome of subsequent pregnancies in patients with a history of peripartum cardiomyopathy. *European Journal of Heart Failure*.

[14] McMurray J. J. V., Packer M., Desai A. S. (2014). Angiotensin-neprilysin inhibition versus enalapril in heart failure. *The New England Journal of Medicine*.

